# A Review on Regenerating Materials from Spent Lithium-Ion Batteries

**DOI:** 10.3390/molecules27072285

**Published:** 2022-03-31

**Authors:** Rui Xu, Wei Xu, Jinggang Wang, Fengmei Liu, Wei Sun, Yue Yang

**Affiliations:** 1 School of Minerals Processing and Bioengineering, Central South University, Changsha 410083, China; 18370956193@163.com (R.X.); cu18535601121@163.com (J.W.); 2 Quzhou Huayou Cobalt New Material Co., Ltd., Quzhou 324002, China; alinkor@huayou.com (W.X.); lfm@huayou.com (F.L.); 3 Key Laboratory of Hunan Province for Clean and Efficient Utilization of Strategic Calcium-Containing Mineral Resources, Central South University, Changsha 410083, China

**Keywords:** spent lithium-ion battery, cathode materials, regeneration of functional materials

## Abstract

Recycling spent lithium-ion batteries (LIBs) have attracted increasing attention for their great significance in environmental protection and cyclic resources utilization. Numerous studies focus on developing technologies for the treatment of spent LIBs. Among them, the regeneration of functional materials from spent LIBs has received great attention due to its short process route and high value-added product. This paper briefly summarizes the current status of spent LIBs recycling and details the existing processes and technologies for preparing various materials from spent LIBs. In addition, the benefits of material preparation from spent LIBs, compared with metals recovery only, are analyzed from both environmental and economic aspects. Lastly, the existing challenges and suggestions for the regeneration process are proposed.

## 1. Introduction

**Figure 1:**
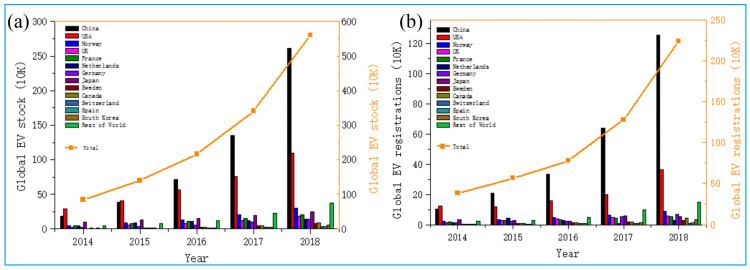
The development of EVs in the world: (**a**) global EV reserves; (**b**) global EV registrations. Adapted with permission from Ref. [1]. Copyright 2018, the Elsevier.

The demand for lithium-ion batteries (LIBs) has continued to grow due to the development of new energy vehicles. According to data from the International Energy Agency on electric vehicle inventories and new registrations by selected countries, the overall trend increased in a straight line clearly from 2014 to 2018 (Figure 1). The number of global LIBs electric vehicles has reached more than 5,000,000 vehicles in 2018 [1]. The rapid development of electric vehicles requires a large number of LIBs raw materials. For example, regarding lithium, an investigation of the Summary of United States Geological Survey mineral products in 2018 reveals that the proven reserves of lithium in the earth’s crust exceed 53 million tons [2]. Economically, only about 30% of this, just about 16 million tons, is worth mining [3]. It is predicted that lithium consumption will increase from 87,400 tons in 2016 to 246,100 tons in 2025, representing a composition annual growth rate (CAGR) of 12.19%. As the main object of lithium consumption, the LIB industry will increase from 45,000 tons in 2016 to 138,000 tons in 2025, the CAGR reaching 11.85% [4]. The imbalance between the supply and demand of raw materials has become a bottleneck problem that is restricting the development of the LIB industry.

On the other hand, the development of the LIB industry will produce a mass of spent LIBs. In spent LIBs, the proportion of cathode, anode, and electrolyte is about 33:10:12 [5]. If not properly treated, the impact of the heavy metals such as lithium, cobalt, manganese, and nickel in the cathode material, as well as electrolyte and its residual harmful components such as HF, PF_5,_ and alkane, on soil, water, and atmospheric environment is immeasurable [6]. Therefore, recycling valuable metals from spent LIBs is of great ecological and environmental importance. It can alleviate the shortage of valuable metals (lithium, nickel, cobalt, etc.) and accelerate the long-term development of the LIB industry, and also protect the environment [7].

**Figure 2:**
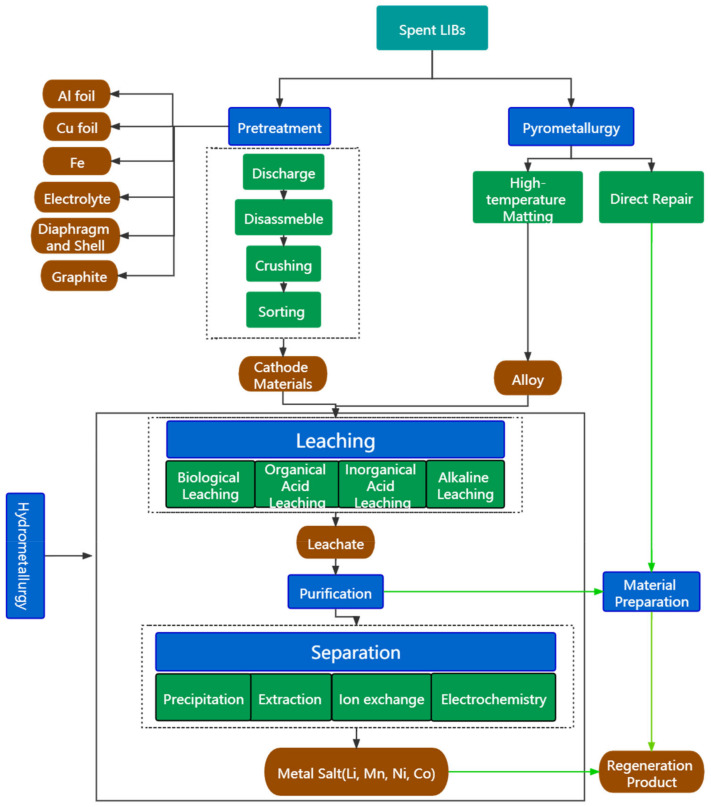
Flowchart for recycling spent LIBs.

The traditional methods for recycling spent LIBs are usually categorized as pretreatment, pyrometallurgy, and hydrometallurgy [8]. The pretreatment for recycling spent LIBs (Figure 2) generally includes discharge, disassembly, and physical sorting (e.g., magnetic separation, gravity separation, etc.). Discharging is the first step for pretreatment. Salt solution of NaCl or MnSO_4_ has been widely used for discharging [4]. It is also found that FeSO_4_ solution is a more environmentally friendly medium than NaCl and MnSO_4_ [9]. The results showed that the active discharge time (ADT) becomes long, and the discharge platform is unstable as the concentration of MnSO_4_ increases. The minimum discharge charge and ADT of FeSO_4_ solution are slightly lower than NaCl. The discharge products of FeSO_4_ are inorganic components, mainly including N_2_, H_2_O, H_2,_ and CO_2_. However, due to the strong corrosion of NaCl, there is electrolyte leakage in the discharge process. The pollutants produced by NaCl solution mainly include hydrocarbons (CH_4_, C_2_H_4_, C_2_H_6_, C_2_H_8_, C_3_H_6_, C_3_H_8_, C_4_H_10_), CH_3_OCH_3_, and CH_3_OCOOCH_3_. Salt solution discharge is one way that can ensure complete discharge and avoid causing a short-circuit explosion by overheating, but the reaction requires a long time. To improve discharge efficiency, external resistance such as metal powder or graphite can be added to promote short circuits. After the discharge process, the spent LIBs are separated into anode and cathode materials, electrolytes, etc. The crushing process opens and dissociates the spent LIBs [10]. The magnetic separation preliminary removes the iron shell, and the winnowing removes the separator [10]. A critical component in the sorting process is wiping off the organic binder from the current collector. The most common approach is solvent extraction by *N*-methylpyrrolidine (NMP), G-butyrolactone, dimethylformamide, and dimethyl sulfoxide [11,12]. In addition, thermal treatment has been proved to be another effective way for binder removal [13]. It has a simple operation and short process, and it provides industrial, large-scale applications. However, it just simply divides the products into anode and cathode materials, Al and Cu foils, etc. [14,15]. The recycling of spent cathode and anode materials needs further processing. The recovery of anode materials mainly involves the separation and reuse of graphite [16]. The conventional recovery process of spent LIB cathode materials is the pyrometallurgy process [17]. The spent LIBs are calcined and reduced by high temperature, and some metals such as manganese, cobalt, and nickel turn to the molten phase as alloy [18]. However, the pyrometallurgy method consumes massive amounts of energy and easily causes air pollution [19]. More importantly, lithium tends to disperse in gas and slag, resulting in the low recovery efficiency of lithium. Compared with pyrometallurgy, the hydrometallurgy method can achieve high metal recovery [20]. The hydrometallurgy method is the most widely used method for recovering spent cathode metals. In this process, cathode materials are leached in different leaching agents such as inorganic acid, organic acid, and alkali liquor [21]. The leachate is separated by extraction, i.e., precipitation purification. The recycled products are metallic salt solutions such as Li_2_SO_4_, Li_2_CO_3_, Ni_2_SO_4_, etc. However, the process is complex, the value-added of protection is low, and many variables need to be controlled.

**Figure 3:**
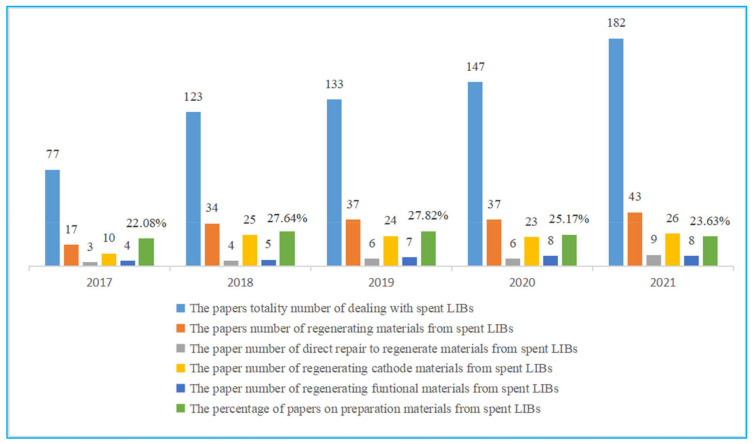
The percentage of papers on preparation materials from recycling spent LIBs in the last five years.

Although great progress has been made in recycling spent LIBs by hydrometallurgy and pyrometallurgy processes, it still faces problems such as long processes, low-value-added products, and serious pollution [22]. In recent years, a new novel idea on the purpose of regenerating functional materials from spent LIBs has been proposed. The regeneration of materials is achieved in two ways. One is to directly repair the cathode materials from spent LIBs, while the other is to employ hydrometallurgy leachate as raw material in resynthesized materials [23]. As can be seen in the green lines in Figure 2, this idea avoids a complex separation process, reduces the emission of pollution gas, and obtains high-value-added products. Therefore, it has attracted extensive attention from academia and industry. We calculated the number of papers related to the preparation of functional materials from spent LIBs; the total number of papers dealing with spent LIBs has grown from 77 to 182 (Figure 3). The percentage of papers on preparation materials from spent LIBs has increased from 22.08% in 2017 to 23.63% in 2022. And the number of studies in each country in the references is counted (Table 1). The number of research results on regenerating materials from spent LIBs was found to be higher. In this paper, different technologies of material regeneration—namely, direct repair, regenerating cathode materials, and regenerating functional materials—from spent LIBs in the last five years is summarized. In addition, the economic and environmental impacts of these technologies are also assessed. By summarizing and analyzing the latest progress, we hope to discover the current problems in the regeneration of materials from spent LIBs and look forward to the development of material regeneration from spent LIBs in the future.

## 2. Technologies for Material Regeneration

### 2.1. Regeneration of Cathode Materials

#### 2.1.1. Direct Repair 

**Figure 4:**
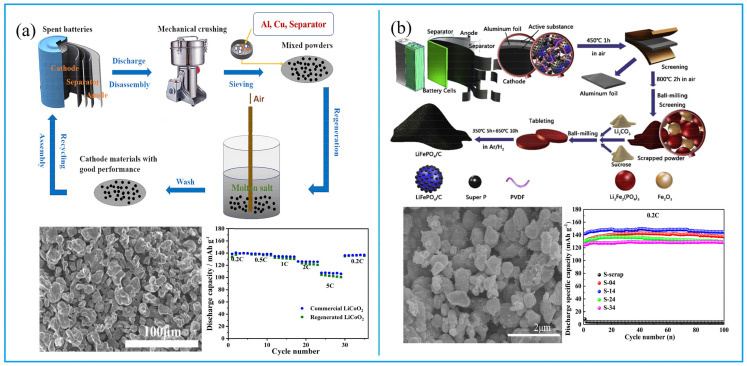
(**a**) The process and characteristics of direct repair LiCoO_2_. Adapted with permission from Ref. [25]. Copyright 2021, the Elsevier Science; (**b**) the process and performances of directly repaired LiFePO_4_. Adapted with permission from Ref. [29]. Copyright 2019, the Elsevier Science.

During the charging and discharging process of LIBs, the performance of the cathode material is often reduced due to the lack of lithium or the collapse of the crystal lattice [24]. The direct repair technology of cathode materials is to restore the electrochemical performance of the material by adding lithium or recrystallization. Yang et al. directly heated spent LiCoO_2_ at 500 °C for 16 h in LiOH-KOH fundamental salt with 0.05 mol LiNO_3_ in an aluminum oxide crucible, and the regenerated LiCoO_2_ can be obtained, the morphology of regenerated material is clearly granular, with good rate capability performance (Figure 4a) [25]. Li et al. studied the recovering process of LiCoO_2_ by adopting different lithium salts as lithium sources, which is used to change the Li/Co ratio in cathode materials [26]. They concluded that Li_2_CO_3_ is the best option, and the compensation of the Li source occurs in the calcination at the temperature of 600–800 °C. The results implied that the first discharge capacity of regenerated cathode materials is 160 mAh/g at 0.2 C, between 3.0 and 4.3 V. Zhang et al. mixed the spent cathode materials with Li_2_CO_3_ in the solution and renovated the LiCoO_2_ with ultrasound radiation under the optimal reaction conditions of 120 °C and ultrasonic power of 999 W, for 10 h. The discharge capacity of repaired LiCoO_2_ reached 132.6 mAh/g at 1 C, with a high capacity retention of 98.1% after 50 cycles at 1 C [27]. Kim et al. also renovated LiCoO_2_ using a hydrothermal method in a concentrated LiOH solution at 200 °C [28]. It was found that the repaired LiCoO_2_ showed the first discharge capacity of 144.0 mAh/g at 0.2 C and a discharge capacity retention of 92.2% after 40 cycles at 0.2 C.

Similar to LiCoO_2_, spent LiFePO_4_ can also be repaired by supplementing lithium. In a study by Sun et al., spent LiFePO_4_ scrap was heated with sucrose and Li_2_CO_3_ at 650 °C for 10 h under Ar/H_2_ in the tube furnace. The repaired cathode materials showed good cycling performance (Figure 4b). The morphology of the regenerated LiFePO_4_/C is spinel olive type. The repaired cathode materials under different situations can obviously restore the electrochemical cycling performance [29]. It was also found that different calcining temperatures can significantly influence electrochemical performances by influencing particles’ agglomeration. A temperature between 650 °C and 700 °C has proved to be the best condition for regenerating cathode material of LiFePO_4_ [30,31]. 

In summary, the direct repair method has the advantages of having a short process and simple operation. However, it is difficult to recycle the complex and mixed spent cathode materials.

#### 2.1.2. Materials Regeneration

(1) Cathode Material Regeneration for LIBs

**Figure 5:**
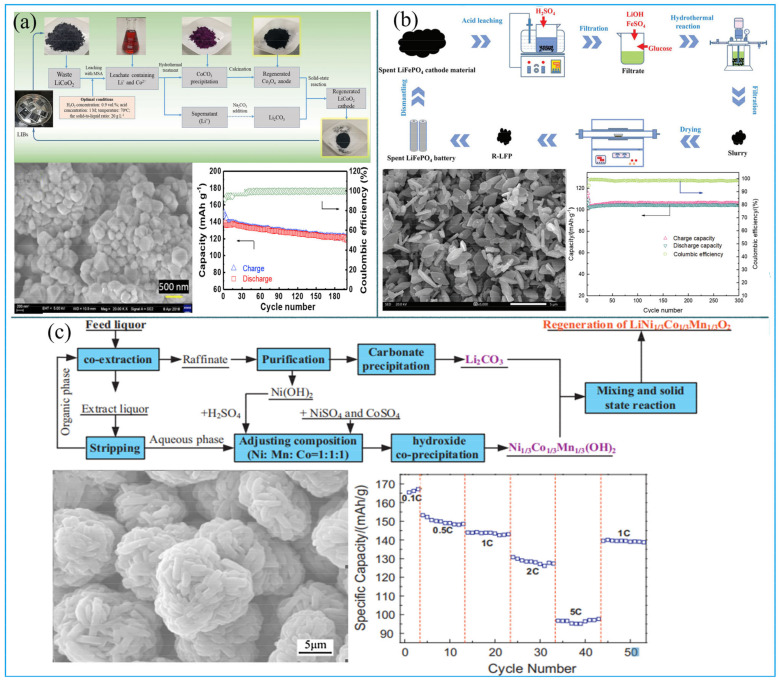
(**a**) The process and characteristics of regenerated LiCoO_2_ cathode materials. Adapted with permission from Ref. [32]. Copyright 2019, the Elsevier Science; (**b**) the process and characteristics of regenerated LiFePO_4_ cathode materials Adapted with permission from Ref. [33]. Copyright 2021, the Royal Society of Chemistry; (**c**) the process and characteristics of regenerated Li(Ni_1/3_Co1_/3_Mn_1/3_) cathode materials. Adapted with permission from Ref. [34]. Copyright 2017, the Elsevier Science.

In cathode material regeneration, leaching solutions are directly converted to regeneration functional materials by adjusting the proportion of elements. For example, the leachate of spent LiCoO_2_ can first be precipitated with Na_2_CO_3_, and then the precipitated CoCO_3_ is further calcined to yield Co_3_O_4_. Finally, new LiCoO_2_ can be generated by solid-phase calcination of a mixture of recovered Co_3_O_4_ and Li_2_CO_3_ (Figure 5a) [32].

Similar technology can also be used to process spent LiFePO_4_ and ternary cathode material. Song et al. regenerated the LiFePO_4_ cathode material from spent LIBs by leaching and hydrothermal method. The authors found that Li^+^ and Fe^2+^ can be leached by H_2_SO_4_ under the antioxidant protection with ascorbic acid. By adjusting the molar ratio of Li:Fe:P in leachate to 3:1:1, the solution was further treated using a hydrothermal method to obtain the LiFePO_4_ cathode material. The discharge capacity of regenerated LiFePO_4_ was 136 mAh/g at 0.1 C, and the retention ratio reached 98.6% at 1 C after 300 cycles (Figure 5b). It was implied that the material is comparable to the commercial LiFePO_4_ cathode material [33]. Yang et al. used H_2_SO_4_ and H_2_O_2_ to leach valuable metals from spent ternary LIBs and then adjusted the molar ratio of metal to regenerate the precursors Ni_1/3_Co_1/3_Mn1_/3_(OH)_2_ by co-precipitation. The precursors were mixed with Li_2_CO_3_ in the muffle furnace and sintered to yield cathode materials. The regeneration ternary cathode material showed electrochemical performances with discharge capacities of 150 mAh/g at 0.5 C and a retention ratio of 96.3% at 1 C, after 50 cycles (Figure 5c) [34]. 

Cathode material regeneration avoids separating valuable metal ion solutions individually from the leaching solution of spent cathode materials. It has a short process and can directly prepare high-value-added materials, which has excellent application prospects.

(2) Other Function Materials

In addition to the regeneration of LIBs materials, the preparation of other functional materials, such as catalysts, sorbents, magnetic materials, etc., has gradually received attention. At present, the process of regenerating functional materials from spent LIBs is similar to that of regenerating cathode material. Different methods, such as the sol–gel method, oxidation–reduction method, and hydrothermal treatment method, can be adopted according to the different synthesis functional materials [35].

**Figure 6:**
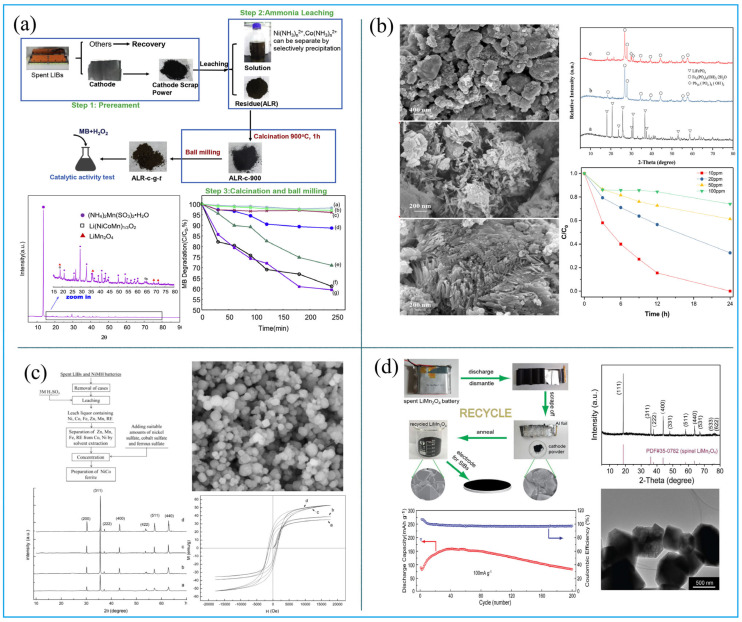
Images of different functional materials: (**a**) catalyst (adapted with permission from Ref. [37]. Copyright 2019, the Elsevier Science); (**b**) sorbent (adapted with permission from Ref. [39]. Copyright 2019, the Elsevier Science); (**c**) ferrite (adapted with permission from Ref. [41]. Copyright 2015, the Elsevier Science); (**d**) miscellaneous recycled materials from spent LiMnO_4_ (adapted with permission from Ref. [43]. Copyright 2019, the Elsevier Science).

Catalysts. Manganese-based catalysts synthesized from spent LIBs have induced a worldwide interest due to their catalytic performance in degrading organic volatiles. The regenerated manganese-based catalyst materials always have the advantages of large specific surface area, abundant mesoporous structure, and Mn^4+^/Mn^3+^ [36]. Shen et al. investigated the manganese-based catalysts Mn_3_O_4_ and NiMn_2_O_4_ that recovered from the ammonia leachate of spent LIBs [37]. When used for removing the methylene blue (MB), the degradation rate increased to 40% from 11% (Figure 6a). Guo et al. obtained a manganese-based perovskite catalyst from spent LIBs by the sol–gel method, and the regenerated manganese-based perovskite catalyst exhibited better catalyst performance for removing toluene than the pure manganese perovskite catalyst [38]. They also discovered that other metal ions in the cathode materials such as Li, Al, Cu, Ni, and Co affect the catalyst properties. It was indicated that the Li and Al suppress the conversion of volatile organic chemicals (VOCs), while the other ions facilitate the reaction rate. 

Sorbents. Nascimento et al. synthesized polymetallic nanoparticles that were composed of Co, Ni, Mn, and Cu from spent LIBs by acid leaching and chemical reduction with NaBH_4_. As the recycled polymetallic nanoparticles had porous and loose spherical surfaces, the dye adsorption efficiency was up to 73%. Xu et al. recovered iron hydroxyl phosphate composites (FPOH) from spent LiFePO_4_ batteries by using hydrothermal treatment. Their results showed a high adsorption rate of Pb, and the maximum adsorption capacity was 43.203 mg/g. In addition, the FPOH composites could also entirely degrade methylene blue in 24 h (Figure 6b) [39]. 

Ferrite. Ferrite has excellent characteristics of magnetism, chemical properties, and material structure and is therefore applied in biomedical, electronic, and recording technologies [40]. Xi et al. prepared the Ni-Co ferrite from the spent LIBs and waste nickel–metal hydride batteries by using the sol–gel combustion method [41]. They discovered that the regenerated ferrite has excellent magnetism of saturation magnetization (Ms), with a value of 52.967 emu/g, while remanent magnetization (Mr) was 25.065 emu/g, and corresponding coercivity (Hc) was 1484.2 Oe (Figure 6c). Rocha et al. manufactured copper ferrite (CuFe_2_O_4_-LiB) from spent LIBs by precipitation. This method is used in the Fenton process to decolorize methylene blue [42]. The decolonization rate of methylene blue could reach 96.1%.

Miscellaneous. Nie et al. reported that reclaimed LiMnO_4_ by thermal treatment from spent LIBs as cathode material for sodium batteries. The regenerated material showed a good coulombic efficiency (Figure 6d) [43]. Li et al. regenerated the spinel lithium ion-sieve (Li_1.6_Mn_1.6-x_Fe_x_O_4_ or LMO) by hydrothermal method from spent LIBs. It was discovered that the LMO has the advantage of faster ion diffusion. Therefore, it can be used to synthesize the lithium-ion sieve, to extract Li from seawater and salt lake brine [44]. Cheng et al. synthesized MnO_2_–NiCo_2_O_4_ anode material with a sea-urchin-like structure from spent LiNi_0.6_Co_0.2_Mn_0.2_O_2_. Additionally, the excellent performances verified the rationality of the method [45].

Regeneration of functional materials from spent LIBs is a potential application direction. In the application of catalysis and environmental protection, in particular, it has achieved the purpose of treating waste with waste [46].

## 3. Environmental and Economic Analysis

### 3.1. Environmental Impact 

**Figure 7:**
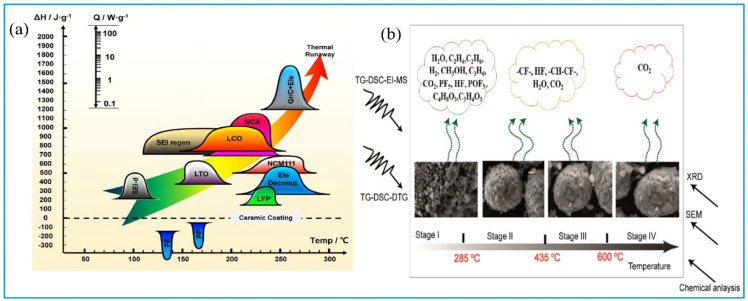
(**a**) The thermal runaway mechanism of different types of spent LIBs (adapted with permission from Ref. [51]. Copyright 2019, the Elsevier Science); (**b**) the main pyrolysis gases during the different temperatures (adapted with permission from Ref. [52]. Copyright 2019, the American Chemical Society).

As mentioned above, compared with the recovery of valuable metals, regenerating materials have the advantage of obtaining value-added products. However, attention should be paid to secondary pollution [47]. The main sources of pollution result from the different processes involved, including the pretreatment process, heat treatment, leaching process, and material regeneration process [14]. During the pretreatment process, comprising discharge, crushing, and electrolyte recovery, it produces waste brine from the salt solution required for discharge [48]; solid waste from steel shell, aluminum foil, copper foil, diaphragm, etc.; exhaust gas from the volatilization of harmful components of the electrolyte [49]. In the process of heat treatment, thermal runaway is the main process of gas pollution. According to the mechanism of the thermal runaway reaction, the battery components undergo a chain reaction in the reduction atmosphere [50]. Different types of battery cathode materials have different reaction temperatures (Figure 7) [51]. Chen et al. analyzed the different types of gases produced by pyrolysis at different temperatures comprehensively [52]. There are mainly alkane, fluoride, carbon monoxide, carbon dioxide, hydrogen fluoride, and other toxic and flammable explosive gases, which come mainly from electrolytes and binders such as C_3_H_4_O_3_ (ethylene carbonate), C_4_H_8_O_3_ (ethyl methyl carbonate) and LiPF_6_ [53]. During the leaching and material regeneration processes, the main pollution is wastewater; however, wastewater can be reused after some proper processing [54]. 

**Figure 8:**
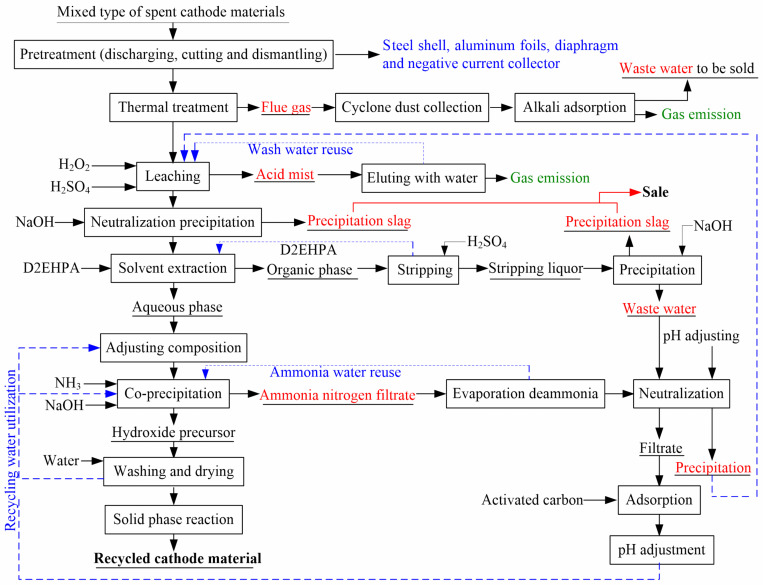
The flow of whole production process, pollution nodes, and the corresponding treatment method. Adapted with permission from Ref. [54]. Copyright 2018, the Elsevier Science.

Although these environmental problems still exist, they can be prevented by effective means. Regarding exhaust gas, fluoride and carbon dioxide generated by pyrolysis can be recovered and utilized by alkali liquor [55]. The acid mist is usually eluted by water, and the acid washing water can be used in the leaching process [54]. The water used for filtration and washing can be recycled in the process (Figure 8). The residual active materials of spent LIBs in different processes were mainly composed of carbon powder, fluorocarbon, and hydrocarbons that were not destroyed after the pyrolysis; thus, they can be reused as raw materials for producing new LIBs [56]. The shell, Al foil, and Cu foil can be recovered by reusing all kinds of materials [57]. On the other hand, the main difference between the only recyclable valuable metals and the regeneration of renewable materials lies in whether the leaching solution needs to separate various metal ions one by one. The recovery of valuable metals requires the use of different leaching agents, extraction agents [58], and other chemical agents, which are prone to a variety of complex chemical reactions. However, the direct preparation of functional materials reduces the leaching and extraction processes, and a single reagent is relatively easier to handle and has less impact on the environment [59]. At the same time, the preparation of cathode materials from raw sulfates also produces a large amount of wastewater as a result of the regeneration of materials from spent LIBs. Therefore, directly recycled materials do not require element separation one by one, which is more environmentally friendly.

### 3.2. Economic Aspects

**Figure 9:**
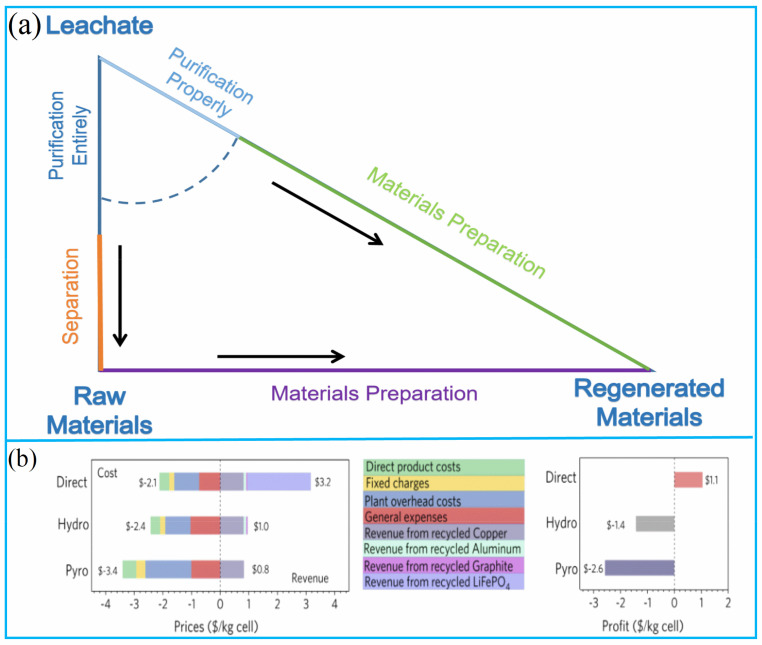
(**a**) Cost comparison chart of different processing methods; (**b**) The potential economic benefit EverBatt model of LiFePO_4_. Adapted with permission from Ref [66]. Copyright 2020, the Elsevier Science.

The cost of conventional recycling of valuable metals and regeneration materials technology from spent LIBs was compared with a triangle chart (Figure 9a). The side length of the triangle represents the cost required for each phase. All pretreatment operations are basically the same between the two technologies of disposal spent LIBs until the leachate is obtained [60]. The first difference is the purification. If recovering valuable metals to be metal salt compounds, deep purification is needed to make the products meet purity standard requirements. However, it is proved that some suitable impurities, such as Al, Cu, etc., can enhance the cathode materials’ properties [61,62,63], so appropriate amounts of impurities are allowed for the regeneration of functional materials from spent LIBs. As a result, the requirements for impurity removal can be appropriately relaxed, and the cost is greatly reduced. The second difference is that the regeneration of materials omits the step of separating different valuable metals one by one, which saves a considerable amount of cost [64]. Therefore, from the point of view of the whole material circulation, the sum of the two sides is greater than the third side (Figure 9a). This means that it is more economical to regenerate materials than to recover valuable metals from spent LIBs under the same conditions of preparation process and cost [65]. Due to the low content of high-value metals in spent lithium iron phosphate, this advantage is particularly evident in the recycling of spent lithium iron phosphate (Figure 9b) [66]. Xu et al. analyzed the potential economic benefits of the EverBatt model developed by Argonne National Laboratory [67]. By the comparison of direct regeneration, pyrometallurgy recycling, and hydrometallurgy, they revealed that only directly regeneration materials are profitable. 

## 4. Conclusions and Perspectives

This paper provided a comprehensive summary of the materials regeneration from spent LIBs. It was indicated that cathode materials, as well as other functional materials, can be regenerated from spent LIBs with environmental and economic benefits. Compared with directly repaired materials, regenerated cathode materials from leaching solutions of spent LIBs are more practical. In addition, regeneration of catalytic materials, adsorbent materials, and other functional materials from spent LIBs also show great application prospects.

Although great progress has been made, there are still some issues that should be addressed [68]. The first one is to develop a direct repair method for spent LIB materials with better raw material adaptability. The second one is the consistency of regenerated materials, which requires stable and reliable processes for regenerating materials and controllable impurity content. The last one is cost and environmental protection—it is important to develop greener and more economical methods for the regeneration of materials. 

## Figures and Tables

**Table 1:** Statistical data about the references in this paper.

The Number of Documents	78
Documents in China	60
Documents in Spain	2
Documents in USA	4
Documents in UK	1
Documents in Germany	2
Documents in Italy	1
Documents in Sweden	1
Documents in Austria	1
Documents in Canada	1
Documents in Brazil	1
Documents in South Korea	1
Documents in the Netherlands	1
Documents in Iran	1
Documents in Japan	1

## Data Availability

Not applicable.

## Abbreviations

Lithium-ion batteries	LIBs
Composition annual growth rate	CAGR
Active discharge time	ADT
*N*-Methylpyrrolidine	NMP
Methylene blue	MB
Volatile organic chemicals	VOCs
Iron hydroxyl phosphate composites	FPOH
Li_1.6_Mn_1.6−x_Fe_x_O_4_	LMO

## References

[1] Huang B., Pan Z., Su X., An L. (2018). Recycling of Lithium-Ion Batteries: Recent Advances and Perspectives. J. Power Sources.

[2] Liao Z., Zhang S., Li K., Zhang G., Habetler T.G. (2020). Review Article A Survey of Methods for Monitoring and Detecting Thermal Runaway of Lithium-Ion Batteries. J. Power Sources.

[3] Zheng X., Zhu Z., Lin X., Zhang Y., He Y., Cao H., Sun Z. (2018). A Mini-Review on Metal Recycling from Spent Lithium Ion Batteries. Engineering.

[4] Li J., Wang G., Xu Z. (2016). Generation and Detection of Metal Ions and Volatile Organic Compounds (VOCs) Emissions from the Pretreatment Processes for Recycling Spent Lithium-Ion Batteries. Waste Manag..

[5] Yang Y., Lei S., Song S., Sun W., Wang L. (2020). Stepwise Recycling of Valuable Metals from Ni-Rich Cathode Material of Spent Lithium-Ion Batteries. Waste Manag..

[6] Lv W., Wang Z., Cao H., Sun Y., Zhang Y., Sun Z. (2018). A Critical Review and Analysis on the Recycling of Spent Lithium-Ion Batteries. ACS Sustain. Chem. Eng..

[7] Chen P., Sun Y., Yang L., Xu R., Luo Y., Wang X., Cao J., Wang J. (2021). Utilization of Metallurgy—Beneficiation Combination Strategy to Decrease TiO2 in Titanomagnetite Concentrate before Smelting. Minerals.

[8] Yu M., Zhang Z., Xue F., Yang B., Guo G., Qiu J. (2019). A More Simple and Efficient Process for Recovery of Cobalt and Lithium from Spent Lithium-Ion Batteries with Citric Acid. Sep. Purif. Technol..

[9] Yao L.P., Zeng Q., Qi T., Li J. (2019). An Environmentally Friendly Discharge Technology to Pretreat Spent Lithium-Ion Batteries. J. Clean. Prod..

[10] Al-Thyabat S., Nakamura T., Shibata E., Iizuka A. (2013). Adaptation of Minerals Processing Operations for Lithium-Ion (LiBs) and Nickel Metal Hydride (NiMH) Batteries Recycling: Critical Review. Miner. Eng..

[11] Song D., Wang X., Nie H., Shi H., Wang D., Guo F., Shi X., Zhang L. (2014). Heat Treatment of LiCoO2 Recovered from Cathode Scraps with Solvent Method. J. Power Sources.

[12] Wang M., Tan Q., Liu L., Li J. (2019). A Low-Toxicity and High-Efficiency Deep Eutectic Solvent for the Separation of Aluminum Foil and Cathode Materials from Spent Lithium-Ion Batteries. J. Hazard. Mater..

[13] Huang Z., Zhu J., Qiu R., Ruan J., Qiu R. (2019). A Cleaner and Energy-Saving Technology of Vacuum Step-by-Step Reduction for Recovering Cobalt and Nickel from Spent Lithium-Ion Batteries. J. Clean. Prod..

[14] Mayyas A., Steward D., Mann M. (2018). The Case for Recycling: Overview and Challenges in the Material Supply Chain for Automotive Li-Ion Batteries. Sustain. Mater. Technol..

[15] Saneie R., Abdollahi H., Ghassa S., Azizi D., Chehreh Chelgani S. (2022). Recovery of Copper and Aluminum from Spent Lithium-Ion Batteries by Froth Flotation: A Sustainable Approach. J. Sustain. Metall..

[16] Liu J., Shi H., Hu X., Geng Y., Yang L., Shao P., Luo X. (2022). Critical Strategies for Recycling Process of Graphite from Spent Lithium-Ion Batteries: A Review. Sci. Total Environ..

[17] Hou H., Li D., Liu X., Yao Y., Dai Z., Yu C. (2018). Recovery of Waste Li Foils from Spent Experimental Li-Anode Coin Cells for LiFePO_4_/C Cathode. Sustain. Mater. Technol..

[18] Li L., Chen R.J., Zhang X.X., Wu F., Ge J., Xie M. (2012). Preparation and Electrochemical Properties of Re-Synthesized LiCoO2 from Spent Lithium-Ion Batteries. Chin. Sci. Bull..

[19] Sun J., Li J., Zhou T., Yang K., Wei S., Tang N., Dang N., Li H., Qiu X., Chen L. (2016). Nano Energy Toxicity, a Serious Concern of Thermal Runaway from Commercial Li-Ion. Nano Energy.

[20] Shuya L., Yang C., Xuefeng C., Wei S., Yaqing W., Yue Y. (2020). Separation and Puri Fi Cation Technology Separation of Lithium and Transition Metals from Leachate of Spent Lithium- Ion Batteries by Solvent Extraction Method with Versatic 10. Sep. Purif. Technol..

[21] Lou P., Guan M., Wu G., Wu J., Yu H., Zhang W., Cheng Q. (2022). Recycle Cathode Materials from Spent Lithium-Ion Batteries by an Innovative Method. Ionics.

[22] Kriston A., Adanouj I., Ruiz V., Pfrang A. (2019). Quantification and Simulation of Thermal Decomposition Reactions of Li-Ion Battery Materials by Simultaneous Thermal Analysis Coupled with Gas Analysis. J. Power Sources.

[23] Liu B., Huang Q., Su Y., Sun L., Wu T., Wang G., Kelly R.M., Wu F. (2019). Maleic, Glycolic and Acetoacetic Acids-Leaching for Recovery of Valuable Metals from Spent Lithium-Ion Batteries: Leaching Parameters, Thermodynamics and Kinetics. R. Soc. Open Sci..

[24] Liang Q., Yue H., Wang S., Yang S., Lam K., Hou X. (2019). Recycling and Crystal Regeneration of Commercial Used LiFePO_4_ Cathode Materials. Electro Acta.

[25] Yang H., Deng B., Jing X., Li W., Wang D. (2021). Direct Recovery of Degraded LiCoO_2_ Cathode Material from Spent Lithium-Ion Batteries: Efficient Impurity Removal toward Practical Applications. Waste Manag..

[26] Li J., Zhong S., Xiong D., Chen H. (2009). Synthesis and Electrochemical Performances of LiCoO_2_ Recycled from the Incisors Bound of Li-Ion Batteries. Rare Met..

[27] Zhang Z., He W., Li G., Xia J., Hu H., Huang J. (2015). Renovation of LiCoO_2_ Crystal Structure from Spent Lithium Ion Batteries by Ultrasonic Hydrothermal Reaction. Res. Chem. Intermed..

[28] Kim D.S., Sohn J.S., Lee C.K., Lee J.H., Han K.S., Lee Y.-I. (2004). Simultaneous Separation and Renovation of Lithium Cobalt Oxide from the Cathode of Spent Lithium Ion Rechargeable Batteries. J. Power Sources.

[29] Sun Q., Li X., Zhang H., Song D., Shi X., Song J., Li C., Zhang L. (2020). Resynthesizing LiFePO_4_/C Materials from the Recycled Cathode via a Green Full-Solid Route. Alloys Compd..

[30] Chen J., Li Q., Song J., Song D., Zhang L., Shi X. (2016). Environmentally Friendly Recycling and Effective Repairing of Cathode Powders from Spent LiFePO_4_ Batteries. Green Chem..

[31] Online V.A., Song X., Hu T., Liang C., Long H.L., Zhou L., Song W., You L., Wu Z.S., Liu J.W. (2017). Direct Regeneration of Cathode Materials from Spent Lithium Iron Phosphate Batteries Using a Solid Phase Sintering Method. RSC Adv..

[32] Wang B., Lin X.Y., Tang Y., Wang Q., Leung M.K.H., Lu X.Y. (2019). Recycling LiCoO_2_ with Methanesulfonic Acid for Regeneration of Lithium-Ion Battery Electrode Materials. J. Power Sources.

[33] Song Y., Xie B., Song S., Lei S., Sun W., Xu R., Yang Y. (2021). Regeneration of LiFePO_4_ from Spent Lithium-Ion Batteries via a Facile Process Featuring Acid Leaching and Hydrothermal Synthesis. Green Chem..

[34] Yang Y., Xu S., He Y. (2017). Lithium Recycling and Cathode Material Regeneration from Acid Leach Liquor of Spent Lithium-Ion Battery via Facile Co-Extraction and Co-Precipitation Processes. Waste Manag..

[35] Guo H.J., Li X.Q., Li X.H., Wang Z.X., Peng W.X., Sun Q.M., Xie J. (2010). Preparation and Electrochemical Properties of Co_3_O_4_/Graphite Composites as Anodes of Lithium Ion Batteries. J. Cent. South Univ. Technol..

[36] Guo M., Li K., Liu L., Zhang H., Guo W., Hu X., Meng X., Jia J., Sun T. (2019). Manganese-Based Multi-Oxide Derived from Spent Ternary Lithium-Ions Batteries as High-Efficient Catalyst for VOCs Oxidation. J. Hazard. Mater..

[37] Shen B., Zhou P., Chen S., Yuan H., Zhu N., Sun T., Lou Z. (2019). Manganese-Based Catalysts Recovered from Spent Ternary Lithium-Ion Batteries and Its Catalytic Activity Enhanced by a Mechanical Method. J. Clean. Prod..

[38] Guo M., Li K., Liu L., Zhang H., Hu X., Min X., Jia J., Sun T. (2019). Resource Utilization of Spent Ternary Lithium-Ions Batteries: Synthesis of Highly Active Manganese-Based Perovskite Catalyst for Toluene Oxidation. J. Taiwan Inst. Chem. Eng..

[39] Xu L., Chen C., Huo J.B., Chen X., Yang J.C.E., Fu M.L. (2019). Iron Hydroxyphosphate Composites Derived from Waste Lithium-Ion Batteries for Lead Adsorption and Fenton-like Catalytic Degradation of Methylene Blue. Environ. Technol. Innov..

[40] Shen J., Pierce J.P., Plummer E.W., Kirschner J. (2003). The Effect of Spatial Confinement on Magnetism: Films, Stripes and Dots of Fe on Cu(111). J. Phys. Condens. Matter.

[41] Xi G., Xu H., Yao L. (2015). Study on Preparation of NiCo Ferrite Using Spent Lithium-Ion and Nickel-Metal Hydride Batteries. Sep. Purif. Technol..

[42] Rocha A.K.S., Magnago L.B., Santos J.J., Leal V.M., Marins A.A.L., Pegoretti V.C.B., Ferreira S.A.D., Lelis M.F.F., Freitas M.B.J.G. (2019). Copper Ferrite Synthesis from Spent Li-Ion Batteries for Multifunctional Application as Catalyst in Photo Fenton Process and as Electrochemical Pseudocapacitor. Mater. Res. Bull..

[43] Nie X.-J., Xi X.-T., Yang Y., Ning Q.-L., Guo J.-Z., Wang M.-Y., Gu Z.-Y., Wu X.-L. (2019). Recycled LiMn_2_O_4_ from the Spent Lithium Ion Batteries as Cathode Material for Sodium Ion Batteries: Electrochemical Properties, Structural Evolution and Electrode Kinetics. Electrochim. Acta.

[44] Li J., Yang X., Fu Y., Huang H., Zhong Z., Wang Y. (2019). Recovery of Fe, Mn, Ni and Co in Sulfuric Acid Leaching Liquor of Spent Lithium Ion Batteries for Synthesis of Lithium Ion-Sieve and Ni_x_Co_y_Mn_1−x−y_(OH)_2_. Hydrometallurgy.

[45] Cheng Y., Guo G., Cheng X., Liu M., Ji J. (2022). Synthesis and Research of MnO_2_–NiCo_2_O_4_ Anode Material from Spent LiNi_0.6_Co_0.2_Mn_0.2_O_2_ Cathodes. Ionics.

[46] Almehmadi F.A., Alqaed S., Mustafa J., Jamil B., Sharifpur M., Cheraghian G. (2022). Combining an Active Method and a Passive Method in Cooling Lithium-Ion Batteries and Using the Generated Heat in Heating a Residential Unit. J. Energy Storage.

[47] Tharumalingam E., Dusseault M.B., Fraser R. (2019). Study of Energy Storage Systems and Environmental Challenges of Batteries. Renew. Sustain. Energy Rev..

[48] Andrey W.G., Sebastian S., René P., Gernot V., Helmar W., Christoph S., Gisela F. (2015). Thermal runaway of commercial 18650 Li-ion batteries with LFP and NCA cathodes-impact of state of charge and overcharge. RSC Adv..

[49] Busche M.R., Adelhelm P., Sommer H., Schneider H., Leitner K., Janek J. (2014). Systematical Electrochemical Study on the Parasitic Shuttle-Effect in Lithium-Sulfur-Cells at Different Temperatures and Different Rates. J. Power Sources.

[50] Larsson F., Bertilsson S., Furlani M., Albinsson I., Mellander B. (2018). Gas Explosions and Thermal Runaways during External Heating Abuse of Commercial Lithium-Ion Graphite-LiCoO_2_ Cells at different Levels of Ageing. J. Power Sources.

[51] Diaz F., Wang Y., Weyhe R., Friedrich B. (2019). Gas Generation Measurement and Evaluation during Mechanical Processing and Thermal Treatment of Spent Li-Ion Batteries. Waste Manag..

[52] Chen Y., Liu N., Jie Y., Hu F., Li Y., Wilson B.P., Xi Y., Lai Y., Yang S. (2019). Toxicity Identification and Evolution Mechanism of Thermolysis-Driven Gas Emissions from Cathodes of Spent Lithium-Ion Batteries. ACS Sustain. Chem. Eng..

[53] Ruffino B., Zanetti M.C., Marini P. (2011). A Mechanical Pre-Treatment Process for the Valorization of Useful Fractions from Spent Batteries. Resour. Conserv. Recycl..

[54] Yang Y., Song S., Jiang F., Zhou J., Sun W. (2018). Short Process for Regenerating Mn-Rich Cathode Material with High Voltage from Mixed-Type Spent Cathode Materials via a Facile Approach. J. Clean. Prod..

[55] Chen Y.G., Wang C.G., Zhang X.Y., Xie D.M., Wang R.S. (2003). Lithium Salts of Heteropolyacid as the Electrolyte of Lithium-Ion Battery. Synth. Met..

[56] Liu W., Zhong X., Han J., Qin W., Liu T., Zhao C., Chang Z. (2018). Kinetic Study and Pyrolysis Behaviors of Spent LiFePO4 Batteries. ACS Sustain. Chem. Eng..

[57] Liu C., Lin J., Cao H., Zhang Y., Sun Z. (2019). Recycling of Spent Lithium-Ion Batteries in View of Lithium Recovery: A Critical Review. J. Clean. Prod..

[58] Peng F., Mu D., Li R., Liu Y., Ji Y., Dai C., Ding F. (2019). Impurity Removal with Highly Selective and efficient. RSC Adv..

[59] Zhang Z., Qiu J., Yu M., Jin C., Yang B., Guo G. (2020). Performance of Al-Doped LiNi1/3Co1/3Mn1/3O2 Synthesized from Spent Lithium Ion Batteries by Sol-Gel Method. Vacuum.

[60] Wu C., Li B., Yuan C., Ni S., Li L. (2019). Recycling Valuable Metals from Spent Lithium-Ion Batteries by Ammonium Sulfite-Reduction Ammonia Leaching. Waste Manag..

[61] Yang L., Ren F., Feng Q., Xu G., Li X., Li Y., Zhao E., Ma J., Fan S. (2018). Effect of Cu Doping on the Structural and Electrochemical Performance of LiNi1/3Co1/3Mn1/3O2 Cathode Materials. J. Electron. Mater..

[62] Liu D., Wang Z., Chen L. (2006). Comparison of Structure and Electrochemistry of Al- and Fe-Doped LiNi1/3Co1/3Mn1/3O2. Electrochem. Acta.

[63] Sa Q., Heelan J.A., Lu Y., Apelian D., Wang Y. (2015). Copper Impurity Effects on LiNi1/3Mn1/3Co1/3O2 Cathode Material. ACS Appl. Mater. Interfaces.

[64] Wang Y., Ma L., Xi X., Nie Z., Zhang Y., Wen X., Lyu Z. (2019). Regeneration and Characterization of LiNi_0.8_Co_0.15_Al_0.05_O_2_ Cathode Material from Spent Power Lithium-Ion Batteries. Waste Manag..

[65] Yi C., Yang Y., Zhang T., Wu X., Sun W., Yi L. (2020). A Green and Facile Approach for Regeneration of Graphite from Spent Lithium Ion Battery. J. Clean. Prod..

[66] Harper G., Sommerville R., Kendrick E., Driscoll L., Slater P., Stolkin R., Walton A., Christensen P., Heidrich O., Lambert S. (2019). Recycling Lithium-Ion Batteries from Electric Vehicles. Nature.

[67] Xu P., Dai Q., Gao H., Lu J., Chen Z., Xu P., Dai Q., Gao H., Liu H., Zhang M. (2020). Report Efficient Direct Recycling of Lithium-Ion Battery Cathodes by Targeted Healing Efficient Direct Recycling of Lithium-Ion Battery Cathodes by Targeted Healing. Joule.

[68] Alqaed S., Almehmadi F.A., Mustafa J., Husain S., Cheraghian G. (2022). Effect of Nano Phase Change Materials on the Cooling Process of a Triangular Lithium Battery Pack. J. Energy Storage.

